# Serum microRNA expression as an early marker for breast cancer risk in prospectively collected samples from the Sister Study cohort

**DOI:** 10.1186/bcr3428

**Published:** 2013-05-24

**Authors:** Ashley C Godfrey, Zongli Xu, Clarice R Weinberg, Robert C Getts, Paul A Wade, Lisa A DeRoo, Dale P Sandler, Jack A Taylor

**Affiliations:** 1 Laboratory of Molecular Carcinogenesis, National Institutes of Health, National Institute of Environmental Health Sciences, 111 T.W. Alexander Drive, Research Triangle Park, NC 27709, USA; 2 Epidemiology Branch, National Institutes of Health, National Institute of Environmental Health Sciences, 111 T.W. Alexander Drive, Research Triangle Park, NC 27709, USA; 3 Biostatics Branch, National Institutes of Health, National Institute of Environmental Health Sciences, 111 T.W. Alexander Drive, Research Triangle Park, NC 27709, USA; 4 Genisphere, LLC, 2801 Sterling Drive, Hatfield, PA 19440, USA

## Abstract

**Introduction:**

MicroRNAs (miRNAs) are small, non-coding, single-stranded RNAs between 18-22 nucleotides long that regulate gene expression. Expression of miRNAs is altered in tumor compared to normal tissue; there is some evidence that these changes may be reflected in the serum of cancer cases compared to healthy individuals. This has yet to be examined in a prospective study where samples are collected before diagnosis.

**Methods:**

We used Affymetrix arrays to examine serum miRNA expression profiles in 410 participants in the Sister Study, a prospective cohort study of 50,884 women. All women in the cohort had never been diagnosed with breast cancer at the time of enrollment. We compared global miRNA expression patterns in 205 women who subsequently developed breast cancer and 205 women who remained breast cancer-free. In addition within the case group we examined the association of miRNA expression in serum with different tumor characteristics, including hormone status (ER, PR, and HER-2) and lymph node status.

**Results:**

Overall, 414 of 1,105 of the human miRNAs on the chip were expressed above background levels in 50 or more women. When the average expression among controls was compared to cases using conditional logistic regression, 21 miRNAs were found to be differentially expressed (P≤.05). Using qRT-PCR on a small, independent sample of 5 cases and 5 controls we verified overexpression of the 3 highest expressing miRNAs among cases, miR-18a, miR-181a, and miR-222; the differences were not statistically significant in this small set. The 21 differentially expressed miRNAs are known to target at least 82 genes; using the gene list for pathway analysis we found enrichment of genes involved in cancer-related processes. In a separate case-case analyses restricted to the 21 miRNAs, we found 7 miRNAs with differential expression for women whose breast tumors differed by HER-2 expression, and 10 miRNAs with differential expression by nodal status.

**Conclusions:**

miRNA levels in serum show a number of small differences between women who later develop cancer versus those who remain cancer-free.

## Introduction

MicroRNAs (miRNAs) are small, non-coding, single-stranded RNAs ranging in size between 18 and 22 nucleotides; they are typically excised from longer, 60- to 110-nucleotide stem-loop precursors [1,2]. miRNAs are involved in fundamental biological processes, including development, differentiation, apoptosis, and proliferation, and are believed to act predominately as post-transcriptional regulators that can either degrade their mRNA targets or repress their translation [3]. A single miRNA may have multiple mRNA targets, and up to 30% of human genes may be regulated by miRNAs [4,5].

Aberrant expression of miRNAs in cancer was initially identified in B-cell chronic lymphocytic leukemia [6], and miRNA dysregulation has been subsequently reported for many tumor types in which, depending on the specific target mRNA(s), they may act either as tumor suppressor genes or as oncogenes [7,8]. In breast cancer, post-diagnosis miRNA levels have been shown to correlate with a number of tumor characteristics, including stage, vascular invasion, proliferative index, and estrogen receptor/progesterone receptor (ER/PR) status [9,10], and may have prognostic value.

miRNAs have recently been found in human serum and plasma, where they appear to be resistant to RNAase degradation and thus relatively stable, even in stored samples [11]. This stability has made miRNAs appealing candidates for epidemiologic studies of stored samples, particularly since miRNA profiling requires only small amounts of serum or plasma [12]. The use of circulating miRNA profiles as potential early-detection cancer markers has generated considerable interest [13-16], although data addressing such application remain sparse. Initial studies have suggested that serum levels of miRNAs may differ between diagnosed cancer cases and controls [17], and several recent case control studies of breast cancer have reported evidence of differential miRNA expression levels in serum [18-21]. These studies have shown little agreement, perhaps because some have measured only a few miRNAs whereas others have used more comprehensive miRNA screens, but with a small number of subjects. None has used samples obtained prior to diagnosis. Use of such prospective samples avoids a number of important potential biases (for example, differential selection and processing of cases and controls or the possibility that the differences observed in case samples are the result of biopsy, cancer treatments, behavioral changes, stress, or other factors experienced by cases but not controls).

Here, we report on a study that prospectively collected serum samples from 205 women who subsequently developed breast cancer and 205 women who remained cancer-free and that used microarrays to comprehensively assess known miRNAs.

## Materials and methods

### Study population

The Sister Study [22] is a prospective cohort study of 50,884 women and was designed to examine the environmental and genetic determinants of breast cancer. The cohort has been previously described [23]; briefly, women from the US or Puerto Rico were eligible to enroll if they themselves had never had breast cancer but had a full or half-sister who had breast cancer. At baseline interview, all participants provided extensive information, including family history, reproductive history, and information about potential risk factors. Informed consent and blood samples were obtained during a home visit. For women who subsequently developed breast cancer, detailed information on diagnosis was collected from medical records and self-report. Pathology reports were abstracted for tumor grade, stage, and other information, including status for ER, PR, and HER-2 (human epidermal growth factor receptor 2) expression. The study was approved by the Institutional Review Board of the National Institute of Environmental Health Sciences, National Institutes of Health, and the Copernicus Group Institutional Review Board.

### Selection of cases and controls

We designed a matched-pair nested case control study. We selected patients who had confirmed invasive breast cancer, who completed enrollment by August 2008, and whose diagnosis occurred within 18 months following blood draw (*n *= 242). We excluded 29 cases who lacked a serum sample or whose sample had integrity issues during collection and shipping and eight cases whose sample had limited volume, leaving 205 cases that are the focus of our study. For each case, a matched control was selected from the 50,884 participants on the basis of the following criteria: no history of cancer (other than non-melanoma skin cancer), having completed enrollment by August 2008, an available blood sample, same race (non-Hispanic white, black, Hispanic, or other), similar age at enrollment (within 5 years), and similar date of blood draw (within 2 months). Three replicate serum samples from three women (nine samples in total) who were not participants in the study but who provided blood samples that were collected and processed in the same manner as Sister Study participants were used to provide technical replicates.

### Assignment to extraction batches and array chip lot

To minimize possible processing and chip lot effects, samples were assigned to processing batches of seven to nine pairs, and batches had similar distributions of age, race, and date of enrollment. For array hybridization, each batch was assigned to one of two different chip lots ('A' and 'B') in a manner designed to ensure a balance of these same characteristics. The nine replicates (described above) were assigned to the same batch and chip lot. Laboratory personnel were blind to case control status and other phenotype information.

### RNA extraction, labeling, and hybridization

Total RNA was extracted in batches by using a Total RNA purification kit (cat. no. 17200; Norgen Biotek Corp., Thorold, ON, Canada). In accordance with the manufacturer's recommendation not to exceed 200 µL per column, 400 µL of total serum from each individual was split into two equal 200-µL aliquots and then processed separately following the manufacturer's recommended protocol for total RNA purification from serum. An on-column DNase digestion was added before sample elution by using an RNase-Free DNase I Kit (cat. no. 25710; Norgen Biotek Corp.), and the two aliquots were subsequently pooled. Fixed volumes rather than fixed amounts of RNA were used in accordance with other studies [24].

Total RNA (8 µL) was directly labeled by using Flash Tag Biotin HSR Labeling kits (cat. no. HSR30FTA; Genisphere, LLC, Hatfield, PA, USA) in accordance with the instructions of the manufacturer. RNA was heated to 80°C for 10 minutes before labeling to inactivate any residual DNase activity. RNA was hybridized for 42 hours to the GeneChip miRNA 2.0 array (cat. no. 901755; Affymetrix Inc., Santa Clara, CA, USA [25]). The GeneChip miRNA 2.0 arrays contain 100% miRBase version 15 coverage of 131 organisms and contain probes for 3,439 human non-coding RNAs (ncRNAs), including 1,105 miRNAs and 2,334 other ncRNAs (including scaRNAs and snoRNAs). The arrays were washed and stained by using standard Affymetrix protocols and scanned by using an Affymetrix GCS 3000 7G Scanner. Feature intensities were extracted by using miRNA 2.0 array library files. Array hybridization and scanning were completed by Precision Biomarker Resources, Inc. (Evanston, IL, USA). The average Spearman correlation coefficient values for three sets of three technical replicates were all above 0.8 (Additional file 1). Array data were deposited into the NCBI Gene Expression Omnibus (GSE44281).

### Replication samples and qRT-PCR

An independent set of 10 women were used to validate selected miRNAs via quantitative reverse transcription-polymerase chain reaction (qRT-PCR). Five women who provided consent and blood samples but who developed breast cancer prior to completing enrollment were selected as cases, along with five controls who also provided consent and blood samples and who were cancer-free but did not complete enrollment. Total RNA was extracted from serum samples of these women as described above with the addition of Synthetic *C. elegans *miScript miRNA Mimic (cat. no. MSY0000010; Qiagen, Valencia, CA, USA). Synthetic cel-39 was spiked-in at a final concentration of 0.25 fmol/µL prior to extraction and used as a PCR normalization control. The RNA concentration, reverse transcription, and pre-PCR steps were carried out in accordance with a previously published protocol [26]. ExoSAP-IT (cat. no. 78250; Affymetrix Inc.) treatment followed by column purification (cat. no. 28004; Qiagen) in accordance with the protocol of the manufacturer was used to purify the pre-PCR product. Individual PCR was run in triplicate by using 1 µL of purified pre-PCR product. The reaction contained the following components: 2x Taqman universal master mix (cat. no. 4324018; ABI, Carlsbad, CA, USA), 1 µM forward primer, 1 µM universal reverse primer, and 0.2 µM probe. The reaction was run on a Bio-Rad CFX 384 Real-Time System (Bio-Rad Laboratories, Inc., Hercules, CA, USA) by using the following parameters: 55°C for 2 minutes, 95°C for 10 minutes, followed by 40 cycles of 95°C for 15 seconds and 55°C for 1 minute. PCR cycle threshold (Ct) values were recorded for each target gene and for normalization controls and were averaged across three independent runs. Primers for miR-222, miR-181a, miR-1825, and miR-18a were custom-ordered from IDT (San Diego, CA, USA) by using previously published sequences [26]. Primers for cel-39 were designed in the same fashion as above and custom-ordered from IDT.

To determine the best candidate miRNA for PCR normalization in our data set, we ran the array expression data from the 47 miRNAs expressed in almost all individuals through the NormFinder software [27]. NormFinder uses a model-based variance estimation approach [28]. Using these results, we selected as a qRT-PCR normalization control miR-1825, which showed one of the highest stability values across the 410 cases and controls and had blood levels that were similar to those of the three target miRNAs. We used the average of miR-1825 and an external spike-in cel-39 control, a strategy shown to be effective for controlling both technical and biologic variability in qRT-PCR assays from serum [17,24]. The efficiency of the four PCR assays (for miR-181a, miR-18a, miR-222, and miR-1825) was similar for all four assays (Additional file 2). Normalized relative expression was based on Ct values and calculated as 1/(Ct_gene_−Ct_norm_).

### Data processing and statistical analysis

miRNA expression intensity values were background-corrected and normalized across arrays by using the robust multichip average method [29]. The intensity data used in all analysis were log (2)-transformed.

For each array, the miRNA probe set signals were compared with the distribution of signals for anti-genomic probes that had matching GC content (miRNA QC Tool, version 1.0.33.0), and in accordance with the recommendation of the manufacturer, Wilcoxon rank-sum test of *P *value of less than 0.06 was used to identify miRNAs above background. Subsequent analysis was restricted to 414 miRNAs that exceeded background levels in at least 50 women. Conditional logistic regression was used to identify differentially expressed miRNA probes between cases and controls for those 414 probes. Because analysis of circulating miRNAs in prospectively collected samples is still exploratory, we - like some other investigators of circulating miRNAs [30,31] - regard these results as descriptive and not as tests of hypotheses and so provide *P *values that are unadjusted for multiple comparisons.

The association between miRNAs and the tumor characteristics of hormone receptor status (ER, PR, and HER-2) and lymph node status was tested in a case-only logistic analysis, in which race was adjusted for. Chip lot and batch were specified as random effect variables. All statistical analyses were performed by using R 2.15.

### Pathway analysis with ingenuity pathway analysis

miRNAs found to be significantly associated with case control status were further analyzed with ingenuity pathway analysis (IPA) [32]. Using IPA's microRNA target filter, we generated a list of predicted mRNA targets for each of the 21 significant miRNAs. The list was then restricted to the mRNAs listed in the IPA database as experimentally verified targets of any of the 21 miRNAs. This mRNA target list was then used to run a canonical pathway analysis.

## Results

### A large number of miRNAs are detected in serum

**Figure 1:**
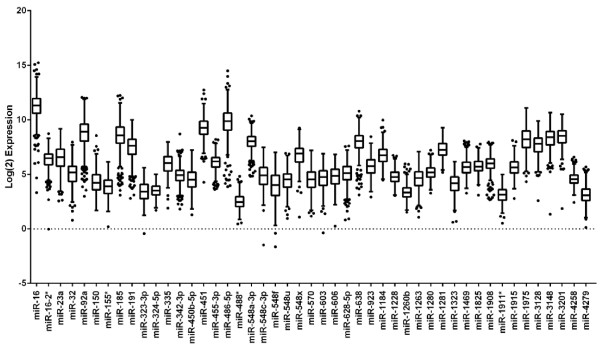
**A large number of microRNAs (miRNAs) are detected in serum**. Box-and-whisker plots showing the log (2)-normalized expression for the 47 miRNAs which are expressed above background in 400 individuals. Expression levels were adjusted for batch and chip lot across all samples. The black line represents the median, and the upper and lower 25% are the top and bottom of the box, respectively. Dots represent the outliers.

In total, 410 serum samples from breast cancer cases (*n *= 205) and controls (*n *= 205) were analyzed in this study; baseline characteristics of the cases and controls are summarized in Table 1. Of the 1,105 human miRNAs, 414 miRNAs were detected above background threshold levels in at least 50 women. Forty-seven miRNAs were detected above background in 400 or more women (Table 2), and miR-16 showed the highest average expression. Even though expression of miRNAs showed considerable inter-individual variation, several miRNAs, including miR-1825 and miR-1228, were relatively constant among women (Figure 1).

**Table 1:** Demographic characteristics of study population

Demographic characteristic	Number of cases	Number of controls
	205	205

Age, years		

40	2	2

40-50	47	47

50-60	64	64

60-70	71	71

≥70	21	21

Race		

White (non-Hispanic)	179	179

Non-white	26	26

**Table 2:** Number of microRNAs detected above background

	Number of microRNAs
In at least 10 individuals	625

In at least 50 individuals	414

In at least 100 individuals	342

In at least 200 individuals	233

In at least 300 individuals	149

In at least 400 individuals	47

### Discovery of differentially expressed miRNAs in serum

When paired case control analysis of the 414 miRNAs expressed above background was used, 21 miRNAs showed significantly different levels in cases and controls (*P *≤0.05) (Table 3). The differences were small, ranging from 4% to 19%. Higher miRNA expression in women destined to become cases was significantly more common (16 of 21 miRNAs) than would be expected by chance alone (binomial test, two-tailed *P *<0.05). Differential miRNA expression was not stronger in women close to their time of diagnosis, but sample size was small and all cases were diagnosed within 18 months of blood draw (data not shown). Using qRT-PCR on a small independent replication set of five cases and five controls, we further examined the three miRNAs (miR-18a, miR-181a, and miR-222) with the highest expression in cases. As predicted, all three miRNAs showed higher levels in cases, although none was statistically significant in this small set of women (Additional file 3).

**Table 3:** Twenty-one differentially expressed microRNAs with a *P *value of not more than 0

Expression	microRNA	*P *value^a^	Percentage change	Previous reports in breast cancer tumor or cell lines
Overexpression	miR-1255a	0.00	10	No data
	
	miR-671-3p	0.01	7	No data
	
	miR-1827	0.01	14	No data
	
	miR-222	0.02	17	Overexpression [40,58,59]
	
	miR-744	0.02	10	No data
	
	miR-4306	0.03	8	No data
	
	miR-151-3p	0.03	15	Overexpression [60]
	
	miR-130b	0.03	14	Overexpression [61]
	
	miR-363	0.03	11	No data
	
	miR-149^a^	0.03	12	No data
	
	miR-652	0.03	15	No data
	
	miR-320d	0.04	14	No data
	
	miR-18a	0.04	19	Overexpression [62-64]
	
	miR-181a	0.05	15	Overexpression [38,65]
	
	miR-3136	0.05	4	No data
	
	miR-629	0.05	7	Overexpression [61]

Underexpression	miR-548d-5p	0.01	−4	No data
	
	miR-760	0.02	−9	No data
	
	miR-1234	0.03	−9	No data
	
	miR-18b^a^	0.04	−5	Underexpression [63]
	
	miR-605	0.05	−7	No data

^a^Conditional logistic regression was used to test for differential gene expression in 414 microRNAs (miRNAs) expressed above background.

### The impact of miRNA alterations on regulatory pathways

To explore potential biological associations, we ran IPA on the 82 experimentally verified mRNA targets of the 21 differentially expressed miRNAs. Sixteen IPA canonical pathways, including molecular mechanisms of cancer, were enriched as were other cancer-related pathways, including p53 signaling, cyclins and cell cycle regulation, and Myc-mediated apoptosis signaling (Additional file 4).

### miRNA expression association with tumor characteristics

**Figure 2:**
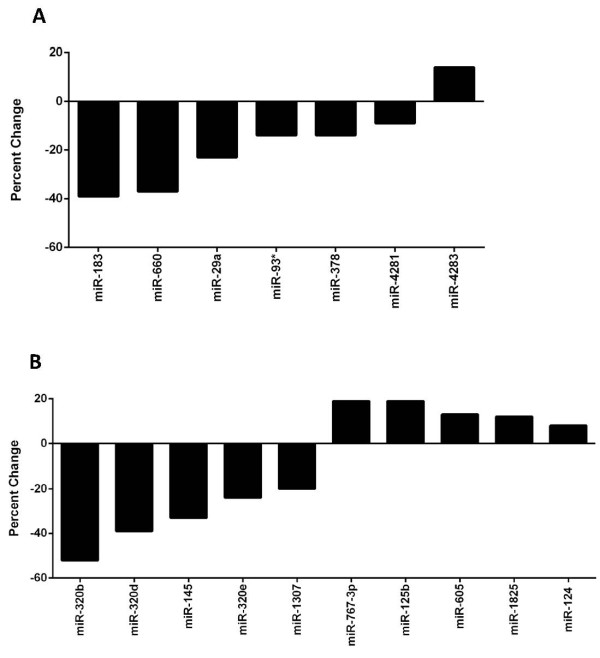
**Serum microRNA (miRNA) expression is associated with tumor subtype**. **(A) **Serum miRNAs significantly associated with HER-2 expression (negative differences correspond to lower levels in women developing tumors with overexpression) (*P *≤0.05). **(B) **Serum miRNAs significantly associated with nodal status (pN1 or higher versus pN0) (*P *≤0.05). *P *values and percentage change were determined by using a linear mixed model.

To investigate the potential association of serum miRNA expression with tumor characteristics in the 205 women who later developed breast cancer, we subclassified them into groups based on tumor characteristics (Table 4) and performed a case-case comparison. There was no evidence of significant differences in serum miRNA levels based on tumor ER or PR staining characteristics. In comparisons of serum samples from the 25 women who developed HER-2-positive tumors with 147 samples from women who developed HER-2-negative tumors, there were seven miRNAs with significantly differential expression (*P *≤0.05); one miRNA was overexpressed and six miRNAs were underexpressed in the HER-2-positive tumors (Figure 2A and Additional file 5). Case-case comparison of serum from women who subsequently developed lymph node-negative tumors (pN0, n = 153) with that of women who developed lymph node-positive tumors (pN1, pN2, or pN3, n = 52) revealed 10 differentially expressed miRNAs (*P *≤0.05); five were overexpressed and five were underexpressed in node-positive tumors (Figure 2B and Additional file 6).

**Table 4:** Patient tumor characteristics

Tumor characteristic	Number	Percentage
Tumor classification		

T1	141	68.78

T2	45	21.95

T3	5	2.44

TX	14	6.83

Lymph node status		

pN0	153	74.63

pN1	38	18.54

pN2	9	4.39

pN3	4	1.95

pNX	1	0.49

Metastasis		

None	202	98.54

Any	3	1.47

Stage		

I	117	57.07

II	59	28.78

III or higher	13	6.34

Could not stage	16	7.80

Estrogen receptor-alpha		

ERα^+^	165	80.49

ERα^−^	37	18.05

Missing	3	1.46

Progesterone receptor		

PR^+^	138	67.32

PR^−^	61	29.76

Missing	6	2.93

HER-2/NEU		

No overexpression	147	71.71

Overexpression	25	12.20

Missing	33	16.10

Breast cancer staging in the Sister Study used a modification of TNM 7 [66].

## Discussion

miRNA profiles are gaining interest as potential diagnostic or prognostic markers for breast cancer [33]. However, existing studies have been limited by sample size or the number of miRNAs analyzed, and none has used prospectively collected samples [18,31,34]. Our study minimized potential biases by profiling global serum miRNA expression patterns in samples obtained from women prior to clinical diagnosis (mean time to diagnosis was 10 months). We found a set of 21 miRNAs differentially expressed in serum samples from 205 women who subsequently developed breast cancer compared with 205 women who remained cancer-free during the time of follow-up. The differences in miRNA levels were small and include both overexpression and underexpression of miRNAs in the cases, and overexpression was significantly more frequent than would be expected had the association been random. Published reports of primary breast tumors or cell lines have examined seven of the 21 differentially expressed miRNAs we found, and all seven showed agreement with the direction of change in our case serum samples (Table 3). IPA of the mRNA targets of these differentially expressed miRNAs suggested gene enrichment for cancer-related signaling pathways. Although the absolute differences in miRNA levels between serum samples of cases and controls are quite small, differences pre-date clinical diagnosis and may reflect important pathways for breast cancer development.

miR-18a, miR-181a, and miR-222 showed the highest percentage difference between cases and controls in our study; qRT-PCR of these miRNAs in a small independent replication set of cases and controls, though not statistically significant, replicated the direction of change for all three. These three miRNAs have been suggested to act as oncogenes through regulation of their potential target mRNAs. miR-18a is part of the oncogenic miR 17-92 cluster, which is often overexpressed in solid tumors, including breast [35]. Overexpression of this cluster is believed to cooperate with c-Myc in stimulating proliferation by negatively regulating E2F1 [36,37]. Increased expression of miR-181a in the bone marrow of patients with breast cancer has been reported to be associated with shorter disease-free survival, higher grade, and breast cancer recurrence [38]. miR-181a is believed to target the tumor suppressor gene programmed cell death protein 4 (*PDCD4*) [38], which inhibits tumor neoplastic transformation [39]. In breast cancer cell lines, miR-222 overexpression has been reported to be associated with tamoxifen resistance through targeting the cell cycle inhibitor p27 (Kip1) [40]. miR-222 has also been reported to increase proliferation of ERα-negative cells while reducing the expression of various tumor suppressor proteins [41], and expression of miR-222 has been reported to increase cell migration in the epithelial-to-mesenchymal transition acting downstream of the RAS-RAF-MEK oncogenic pathway [42].

Interestingly, two recent case control studies have provided evidence that both miR-222 and miR-181a are overexpressed in the serum of patients with breast cancer. One used sequencing by oligonucleotide ligation and detection (SOLiD) of serum samples obtained prior to surgery from 13 breast cancer cases compared with samples from 10 healthy controls and found 26 miRNAs that were overexpressed in cases, including miR-222 and miR-181a; overexpression of miR-222 was validated in an independent group of 50 cases and 50 controls by using qRT-PCR [20]. A second study used Solexa sequencing combined with Taqman low-density array chips on serum samples obtained prior to surgery from 48 breast cancer cases and 48 controls; 10 miRNAs were found to be overexpressed in the cases, and four were validated by using qRT-PCR in an independent group of 76 cases and 76 controls [21]. That study also found overexpression of miR-222 [21]. These studies, combined with our prospective study, provide a growing body of evidence that miR-222 measured in blood is associated with breast cancer.

Among cases, we compared the serum miRNA profiles of women with different tumor characteristics, including hormone status (ER, PR, and HER-2) and nodal status. Although there were no differences in ER or PR status, there were differences in HER-2 and lymph node status. Of the seven miRNAs differentially expressed in the serum of women who developed HER-2-overexpressing breast tumors, miR-93, miR-183, and miR-29a have been reported to be associated with breast cancer in previous studies [20,43,44]. In our study, miR-93 was underexpressed in the serum of women who developed HER-2-overexpressing breast tumors; interestingly, miR-93 expression was recently shown to induce a more differentiated cell phenotype in breast cancer cell lines, and expression of miR-93 in mouse mammary fat pads blocked tumor development and metastases [44]. Of the 10 miRNAs differentially expressed in the serum of women with tumors that spread to the lymph nodes (pN1 or higher), four (miR-145, miR-124, miR-125b, and miR-320) have been reported to be associated with breast cancer in previous studies [45-48]. Of these, miR-320 is of particular interest as we found three miR-320 family members (miR-320b, miR-320d, and miR320e) to be underexpressed in the serum of women who developed lymph node-positive breast tumors. miR-320 has been reported to be decreased in breast tumor tissue and downregulation of miR-320 - through loss of phosphatase and tensin homolog (PTEN) -has been shown to promote tumor proliferation and invasiveness in mouse models; expression of miR-320 distinguished human normal breast stroma from tumor stroma and was correlated with recurrence [49]. A study comparing miRNA expression in inflammatory breast cancer (IBC) with non-IBC also found miR-320 to be downregulated in the more aggressive IBC group of tumors [50]. Thus, loss of miR-320 expression may be associated with a higher likelihood of lymph node involvement and a more aggressive metastatic phenotype.

Although miRNAs that are differentially expressed between tumor and normal tissue are more frequently downregulated in tumor tissue [7], our study (like others [20,21]) has found that circulating miRNAs that differ in levels between breast cancer patients and controls are more frequently at higher levels in case blood samples. The mechanism underlying circulating miRNA stability is still being investigated. One model involves the active release of miRNAs from cells in membrane-bound microvesicles, including exosomes and shedding vesicles [51-53]. There is evidence that microvesicles can deliver miRNAs to recipient cells and trigger changes in target mRNA levels [54]. A recent report has shown that vesicle-encapsulated miRNAs represent only a minor portion of circulating miRNAs but that a significant portion of circulating miRNAs are associated with Argonaute2 (Ago2) [55], the effector component of the miRNA-induced silencing complex [56]. Both models support the possibility that miRNAs may be actively released into circulation and could act as signaling molecules able to regulate their target mRNA expression in recipient cells. Cancer-associated miRNAs in the circulation could also originate from immunocytes in the tumor microenvironment or from a response mediated by the body's systemic response to disease [57].

## Conclusions

We find some evidence of differences in miRNA serum levels between women who subsequently developed cancer compared with women who remained clinically cancer-free. The magnitude of these differences is small, and this may limit their clinical application as circulating early-detection markers for breast cancer. This is the first study to use prospectively collected samples; limitations include a relatively short follow-up period and a sample size that is not large enough to fully explore etiologic versus diagnostic relevance. Our study was carried out within a cohort of women who each had a sister with breast cancer, putting the former at about twofold increased risk, and so the differences that we observed may not be generalizable to women without a similar family history.

## Abbreviations

ER: estrogen receptor; HER-2: human epidermal growth factor receptor 2; IBC: inflammatory breast cancer; IPA: ingenuity pathway analysis; miRNA: microRNA; ncRNA: non-coding RNA; PCR: polymerase chain reaction; PR: progesterone receptor; qRT-PCR: quantitative reverse transcription-polymerase chain reaction.

## Competing interests

The authors declare that they have no competing interests.

## Authors' contributions

ACG carried out sample extraction and sample preparation, did some statistical analysis, and helped to design the experiments, interpret the results, and write the manuscript. ZX performed the majority of the statistical analysis. CRW and PAW participated in study design. LDR participated in study design and provided patient data and study variables. DPS participated in study design and collected samples and data. RCG provided study reagents and processing as well as critical advice on study design, QA, and data interpretation. JAT helped to design the experiments, interpret the results, and write the manuscript. All authors read and approved the final manuscript.

## Supplementary Material

Additional file 1 **Spearman correlation coefficient values for technical replicates of arrays**. Three replicate serum samples from three women (nine samples in total) were processed and hybridized to arrays as described for samples in the main study. Spearman correlation coefficients were calculated for the three pairings of replicate samples for each woman and averaged. One array from Individual 1 appeared to be an outlier but was included in the results shown above. Exclusion of this array resulted in correlation coefficients of greater than 0.97 in all three categories of probes for Individual 1. Click here for file

Additional file 2 **Efficiency of the four polymerase chain reaction (PCR) assays**. The efficiency of PCR amplifications for the normalization control and three target microRNAs (miRNAs) was calculated by using DART-PCR version 1.0. The average efficiency of each of three independent PCRs was similar across all four miRNAs. Click here for file

Additional file 3 **Quantitative reverse transcription-polymerase chain reaction (qRT-PCR) validation in 10 cases and controls**. Serum levels of five cases and five controls were examined by using qRT-PCR for miR181a, miR18a, and miR-222. Box plots show normalized relative expression. Normalization was carried out by using the mean of miR-1825 and spiked in cel-39. The horizontal line represents the mean for each sample. Click here for file

Additional file 4 **Experimentally observed targets enriched for cancer and signaling pathways**. Identified ingenuity pathway analysis (IPA) canonical pathways enriched by the experimentally observed targets of the 21 miRNAs differentially expressed between the cases and non-cases. The negative log (10) false discovery rate-corrected *P *values are shown. Note that this test has not corrected for possible dependencies across the mRNAs considered and that statistical significance may be overstated. HER-2, human epidermal growth factor receptor 2; ILK, integrin-linked kinase; PTEN, phosphatase and tensin homolog. Click here for file

Additional file 5 **List of differentially expressed serum microRNAs (miRNAs) based on HER-2/NEU expression**. Click here for file

Additional file 6 **List of differentially expressed serum microRNAs (miRNAs) based on nodal status**. Click here for file

## References

[B1] Ambros V The functions of animal microRNAs Nature 2004 431 350 355 15372042 10.1038/nature02871

[B2] Pasquinelli AE Hunter S Bracht J MicroRNAs: a developing story Curr Opin Genet Dev 2005 15 200 205 15797203 10.1016/j.gde.2005.01.002

[B3] Sayed D Abdellatif M MicroRNAs in development and disease Physiol Rev 2011 91 827 887 21742789 10.1152/physrev.00006.2010

[B4] Lim LP Lau NC Garrett-Engele P Grimson A Schelter JM Castle J Bartel DP Linsley PS Johnson JM Microarray analysis shows that some microRNAs downregulate large numbers of target mRNAs Nature 2005 433 769 773 15685193 10.1038/nature03315

[B5] Bartel DP MicroRNAs: genomics, biogenesis, mechanism, and function Cell 2004 116 281 297 14744438 10.1016/s0092-8674(04)00045-5

[B6] Calin GA Dumitru CD Shimizu M Bichi R Zupo S Noch E Aldler H Rattan S Keating M Rai K Rassenti L Kipps T Negrini M Bullrich F Croce CM Frequent deletions and down-regulation of micro-RNA genes miR15 and miR16 at 13q14 in chronic lymphocytic leukemia Proc Natl Acad Sci USA 2002 99 15524 15529 12434020 10.1073/pnas.242606799 PMC137750

[B7] Lu J Getz G Miska EA Alvarez-Saavedra E Lamb J Peck D Sweet-Cordero A Ebert BL Mak RH Ferrando AA Downing JR Jacks T Horvitz HR Golub TR MicroRNA expression profiles classify human cancers Nature 2005 435 834 838 15944708 10.1038/nature03702

[B8] Volinia S Calin GA Liu CG Ambs S Cimmino A Petrocca F Visone R Iorio M Roldo C Ferracin M Prueitt RL Yanaihara N Lanza G Scarpa A Vecchione A Negrini M Harris CC Croce CM A microRNA expression signature of human solid tumors defines cancer gene targets Proc Natl Acad Sci USA 2006 103 2257 2261 16461460 10.1073/pnas.0510565103 PMC1413718

[B9] Iorio MV Ferracin M Liu CG Veronese A Spizzo R Sabbioni S Magri E Pedriali M Fabbri M Campiglio M Menard S Palazzo JP Rosenberg A Musiani P Volinia S Nenci I Calin GA Querzoli P Negrini M Croce CM MicroRNA gene expression deregulation in human breast cancer Cancer Res 2005 65 7065 7070 16103053 10.1158/0008-5472.CAN-05-1783

[B10] Mattie MD Benz CC Bowers J Sensinger K Wong L Scott GK Fedele V Ginzinger D Getts R Haqq C Optimized high-throughput microRNA expression profiling provides novel biomarker assessment of clinical prostate and breast cancer biopsies Mol Cancer 2006 5 24 16784538 10.1186/1476-4598-5-24 PMC1563474

[B11] Gilad S Meiri E Yogev Y Benjamin S Lebanony D Yerushalmi N Benjamin H Kushnir M Cholakh H Melamed N Bentwich Z Hod M Goren Y Chajut A Serum microRNAs are promising novel biomarkers PLoS One 2008 3 e3148 18773077 10.1371/journal.pone.0003148 PMC2519789

[B12] Lodes MJ Caraballo M Suciu D Munro S Kumar A Anderson B Detection of cancer with serum miRNAs on an oligonucleotide microarray PLoS One 2009 4 e6229 19597549 10.1371/journal.pone.0006229 PMC2704963

[B13] Weiland M Gao XH Zhou L Mi QS Small RNAs have a large impact: circulating microRNAs as biomarkers for human diseases RNA Biol 2012 9 850 859 22699556 10.4161/rna.20378

[B14] Heneghan HM Miller N Kerin MJ MiRNAs as biomarkers and therapeutic targets in cancer Curr Opin Pharmacol 2010 10 543 550 20541466 10.1016/j.coph.2010.05.010

[B15] Cortez MA Calin GA MicroRNA identification in plasma and serum: a new tool to diagnose and monitor diseases Expert Opin Biol Ther 2009 9 703 711 19426115 10.1517/14712590902932889

[B16] Kosaka N Iguchi H Ochiya T Circulating microRNA in body fluid: a new potential biomarker for cancer diagnosis and prognosis Cancer Sci 2010 101 2087 2092 20624164 10.1111/j.1349-7006.2010.01650.x PMC11159200

[B17] Mitchell PS Parkin RK Kroh EM Fritz BR Wyman SK Pogosova-Agadjanyan EL Peterson A Noteboom J O'Briant KC Allen A Lin DW Urban N Drescher CW Knudsen BS Stirewalt DL Gentleman R Vessella RL Nelson PS Martin DB Tewari M Circulating microRNAs as stable blood-based markers for cancer detection Proc Natl Acad Sci USA 2008 105 10513 10518 18663219 10.1073/pnas.0804549105 PMC2492472

[B18] Asaga S Kuo C Nguyen T Terpenning M Giuliano AE Hoon DS Direct serum assay for microRNA-21 concentrations in early and advanced breast cancer Clin Chem 2011 57 84 91 21036945 10.1373/clinchem.2010.151845

[B19] van Schooneveld E Wouters MC Van der Auwera I Peeters DJ Wildiers H Van Dam PA Vergote I Vermeulen PB Dirix LY Van Laere SJ Expression profiling of cancerous and normal breast tissues identifies microRNAs that are differentially expressed in serum from patients with (metastatic) breast cancer and healthy volunteers Breast Cancer Res 2012 14 R34 22353773 10.1186/bcr3127 PMC3496152

[B20] Wu Q Wang C Lu Z Guo L Ge Q Analysis of serum genome-wide microRNAs for breast cancer detection Clin Chim Acta 2012 413 1058 1065 22387599 10.1016/j.cca.2012.02.016

[B21] Hu Z Dong J Wang LE Ma H Liu J Zhao Y Tang J Chen X Dai J Wei Q Zhang C Shen H Serum microRNA profiling and breast cancer risk: the use of miR-484/191 as endogenous controls Carcinogenesis 2012 33 828 834 22298638 10.1093/carcin/bgs030

[B22] The Sister Study homepage http://www.sisterstudy.org

[B23] D'Aloisio AA Baird DD DeRoo LA Sandler DP Association of intrauterine and early-life exposures with diagnosis of uterine leiomyomata by 35 years of age in the Sister Study Environ Health Perspect 2010 118 375 381 20194067 10.1289/ehp.0901423 PMC2854766

[B24] Kroh EM Parkin RK Mitchell PS Tewari M Analysis of circulating microRNA biomarkers in plasma and serum using quantitative reverse transcription-PCR (qRT-PCR) Methods 2010 50 298 301 20146939 10.1016/j.ymeth.2010.01.032 PMC4186708

[B25] Affymetrix, Inc. homepage http://www.affymetrix.com

[B26] Moltzahn F Hunkapiller N Mir AA Imbar T Blelloch R High throughput microRNA profiling: optimized multiplex qRT-PCR at nanoliter scale on the fluidigm dynamic arrayTM IFCs J Vis Exp 2011 54 2552 21847076 10.3791/2552 PMC3211115

[B27] Molecular Diagnostic Laboratory homepage http://www.mdl.dk/publicationsnormfinder.htm

[B28] Andersen CL Jensen JL Orntoft TF Normalization of real-time quantitative reverse transcription-PCR data: a model-based variance estimation approach to identify genes suited for normalization, applied to bladder and colon cancer data sets Cancer Res 2004 64 5245 5250 15289330 10.1158/0008-5472.CAN-04-0496

[B29] Irizarry RA Hobbs B Collin F Beazer-Barclay YD Antonellis KJ Scherf U Speed TP Exploration, normalization, and summaries of high density oligonucleotide array probe level data Biostatistics 2003 4 249 264 12925520 10.1093/biostatistics/4.2.249

[B30] Chen X Hu Z Wang W Ba Y Ma L Zhang C Wang C Ren Z Zhao Y Wu S Zhuang R Zhang Y Hu H Liu C Xu L Wang J Shen H Zhang J Zen K Zhang CY Identification of ten serum microRNAs from a genome-wide serum microRNA expression profile as novel noninvasive biomarkers for nonsmall cell lung cancer diagnosis Int J Cancer 2012 130 1620 1628 21557218 10.1002/ijc.26177

[B31] Zhao H Shen J Medico L Wang D Ambrosone CB Liu S A pilot study of circulating miRNAs as potential biomarkers of early stage breast cancer PLoS One 2010 5 e13735 21060830 10.1371/journal.pone.0013735 PMC2966402

[B32] Ingenuity Systems, Inc. homepage http://www.ingenuity.com/

[B33] Fu SW Chen L Man YG miRNA biomarkers in breast cancer detection and management J Cancer 2011 2 116 122 21479130 10.7150/jca.2.116 PMC3072617

[B34] Heneghan HM Miller N Lowery AJ Sweeney KJ Newell J Kerin MJ Circulating microRNAs as novel minimally invasive biomarkers for breast cancer Ann Surg 2010 251 499 505 20134314 10.1097/SLA.0b013e3181cc939f

[B35] Song L Lin C Wu Z Gong H Zeng Y Wu J Li M Li J miR-18a impairs DNA damage response through downregulation of ataxia telangiectasia mutated (ATM) kinase PLoS One 2011 6 e25454 21980462 10.1371/journal.pone.0025454 PMC3181320

[B36] He L Thomson JM Hemann MT Hernando-Monge E Mu D Goodson S Powers S Cordon-Cardo C Lowe SW Hannon GJ Hammond SM A microRNA polycistron as a potential human oncogene Nature 2005 435 828 833 15944707 10.1038/nature03552 PMC4599349

[B37] O'Donnell KA Wentzel EA Zeller KI Dang CV Mendell JT c-Myc-regulated microRNAs modulate E2F1 expression Nature 2005 435 839 843 15944709 10.1038/nature03677

[B38] Ota D Mimori K Yokobori T Iwatsuki M Kataoka A Masuda N Ishii H Ohno S Mori M Identification of recurrence-related microRNAs in the bone marrow of breast cancer patients Int J Oncol 2011 38 955 962 21271219 10.3892/ijo.2011.926

[B39] Cmarik JL Min H Hegamyer G Zhan S Kulesz-Martin M Yoshinaga H Matsuhashi S Colburn NH Differentially expressed protein Pdcd4 inhibits tumor promoter-induced neoplastic transformation Proc Natl Acad Sci USA 1999 96 14037 14042 10570194 10.1073/pnas.96.24.14037 PMC24186

[B40] Miller TE Ghoshal K Ramaswamy B Roy S Datta J Shapiro CL Jacob S Majumder S MicroRNA-221/222 confers tamoxifen resistance in breast cancer by targeting p27Kip1 J Biol Chem 2008 283 29897 29903 18708351 10.1074/jbc.M804612200 PMC2573063

[B41] Di Leva G Gasparini P Piovan C Ngankeu A Garofalo M Taccioli C Iorio MV Li M Volinia S Alder H Nakamura T Nuovo G Liu Y Nephew KP Croce CM MicroRNA cluster 221-222 and estrogen receptor alpha interactions in breast cancer J Natl Cancer Inst 2010 102 706 721 20388878 10.1093/jnci/djq102 PMC2873185

[B42] Stinson S Lackner MR Adai AT Yu N Kim HJ O'Brien C Spoerke J Jhunjhunwala S Boyd Z Januario T Newman RJ Yue P Bourgon R Modrusan Z Stern HM Warming S de Sauvage FJ Amler L Yeh RF Dornan D TRPS1 targeting by miR-221/222 promotes the epithelial-to-mesenchymal transition in breast cancer Sci Signal 2011 4 ra41 21673316 10.1126/scisignal.2001538

[B43] Shimono Y Zabala M Cho RW Lobo N Dalerba P Qian D Diehn M Liu H Panula SP Chiao E Dirbas FM Somlo G Pera RA Lao K Clarke MF Downregulation of miRNA-200c links breast cancer stem cells with normal stem cells Cell 2009 138 592 603 19665978 10.1016/j.cell.2009.07.011 PMC2731699

[B44] Liu S Patel SH Ginestier C Ibarra I Martin-Trevino R Bai S McDermott SP Shang L Ke J Ou SJ Heath A Zhang KJ Korkaya H Clouthier SG Charafe-Jauffret E Birnbaum D Hannon GJ Wicha MS MicroRNA93 regulates proliferation and differentiation of normal and malignant breast stem cells PLoS Genet 2012 8 e1002751 22685420 10.1371/journal.pgen.1002751 PMC3369932

[B45] Wu X Somlo G Yu Y Palomares MR Li AX Zhou W Chow A Yen Y Rossi JJ Gao H Wang J Yuan YC Frankel P Li S Ashing-Giwa KT Sun G Wang Y Smith R Robinson K Ren X Wang SE De novo sequencing of circulating miRNAs identifies novel markers predicting clinical outcome of locally advanced breast cancer J Transl Med 2012 10 42 22400902 10.1186/1479-5876-10-42 PMC3342150

[B46] Zhou M Liu Z Zhao Y Ding Y Liu H Xi Y Xiong W Li G Lu J Fodstad O Riker AI Tan M MicroRNA-125b confers the resistance of breast cancer cells to paclitaxel through suppression of pro-apoptotic Bcl-2 antagonist killer 1 (Bak1) expression J Biol Chem 2010 285 21496 21507 20460378 10.1074/jbc.M109.083337 PMC2898411

[B47] Lv XB Jiao Y Qing Y Hu H Cui X Lin T Song E Yu F miR-124 suppresses multiple steps of breast cancer metastasis by targeting a cohort of pro-metastatic genes in vitro Chin J Cancer 2011 30 821 830 22085528 10.5732/cjc.011.10289 PMC4013330

[B48] Yan LX Huang XF Shao Q Huang MY Deng L Wu QL Zeng YX Shao JY MicroRNA miR-21 overexpression in human breast cancer is associated with advanced clinical stage, lymph node metastasis and patient poor prognosis RNA 2008 14 2348 2360 18812439 10.1261/rna.1034808 PMC2578865

[B49] Bronisz A Godlewski J Wallace JA Merchant AS Nowicki MO Mathsyaraja H Srinivasan R Trimboli AJ Martin CK Li F Yu L Fernandez SA Pecot T Rosol TJ Cory S Hallett M Park M Piper MG Marsh CB Yee LD Jimenez RE Nuovo G Lawler SE Chiocca EA Leone G Ostrowski MC Reprogramming of the tumour microenvironment by stromal PTEN-regulated miR-320 Nat Cell Biol 2012 14 159 167 22179046 10.1038/ncb2396 PMC3271169

[B50] Van der Auwera I Limame R van Dam P Vermeulen PB Dirix LY Van Laere SJ Integrated miRNA and mRNA expression profiling of the inflammatory breast cancer subtype Br J Cancer 2010 103 532 541 20664596 10.1038/sj.bjc.6605787 PMC2939785

[B51] Hunter MP Ismail N Zhang X Aguda BD Lee EJ Yu L Xiao T Schafer J Lee ML Schmittgen TD Nana-Sinkam SP Jarjoura D Marsh CB Detection of microRNA expression in human peripheral blood microvesicles PLoS One 2008 3 e3694 19002258 10.1371/journal.pone.0003694 PMC2577891

[B52] Kosaka N Iguchi H Yoshioka Y Takeshita F Matsuki Y Ochiya T Secretory mechanisms and intercellular transfer of microRNAs in living cells J Biol Chem 2010 285 17442 17452 20353945 10.1074/jbc.M110.107821 PMC2878508

[B53] Simons M Raposo G Exosomes--vesicular carriers for intercellular communication Curr Opin Cell Biol 2009 21 575 581 19442504 10.1016/j.ceb.2009.03.007

[B54] Valadi H Ekstrom K Bossios A Sjostrand M Lee JJ Lotvall JO Exosome-mediated transfer of mRNAs and microRNAs is a novel mechanism of genetic exchange between cells Nat Cell Biol 2007 9 654 659 17486113 10.1038/ncb1596

[B55] Arroyo JD Chevillet JR Kroh EM Ruf IK Pritchard CC Gibson DF Mitchell PS Bennett CF Pogosova-Agadjanyan EL Stirewalt DL Tait JF Tewari M Argonaute2 complexes carry a population of circulating microRNAs independent of vesicles in human plasma Proc Natl Acad Sci USA 2011 108 5003 5008 21383194 10.1073/pnas.1019055108 PMC3064324

[B56] Meister G Landthaler M Patkaniowska A Dorsett Y Teng G Tuschl T Human Argonaute2 mediates RNA cleavage targeted by miRNAs and siRNAs Mol Cell 2004 15 185 197 15260970 10.1016/j.molcel.2004.07.007

[B57] Ma R Jiang T Kang X Circulating microRNAs in cancer: origin, function and application J Exp Clin Cancer Res 2012 31 38 22546315 10.1186/1756-9966-31-38 PMC3431991

[B58] Zhao JJ Lin J Yang H Kong W He L Ma X Coppola D Cheng JQ MicroRNA-221/222 negatively regulates estrogen receptor alpha and is associated with tamoxifen resistance in breast cancer J Biol Chem 2008 283 31079 31086 18790736 10.1074/jbc.M806041200 PMC2576549

[B59] Guttilla IK Phoenix KN Hong X Tirnauer JS Claffey KP White BA Prolonged mammosphere culture of MCF-7 cells induces an EMT and repression of the estrogen receptor by microRNAs Breast Cancer Res Treat 2012 132 75 85 21553120 10.1007/s10549-011-1534-y

[B60] Zhang L Huang J Yang N Greshock J Megraw MS Giannakakis A Liang S Naylor TL Barchetti A Ward MR Yao G Medina A O'Brien-Jenkins A Katsaros D Hatzigeorgiou A Gimotty PA Weber BL Coukos G microRNAs exhibit high frequency genomic alterations in human cancer Proc Natl Acad Sci USA 2006 103 9136 9141 16754881 10.1073/pnas.0508889103 PMC1474008

[B61] Navon R Wang H Steinfeld I Tsalenko A Ben-Dor A Yakhini Z Novel rank-based statistical methods reveal microRNAs with differential expression in multiple cancer types PLoS One 2009 4 e8003 19946373 10.1371/journal.pone.0008003 PMC2777376

[B62] Leivonen SK Makela R Ostling P Kohonen P Haapa-Paananen S Kleivi K Enerly E Aakula A Hellstrom K Sahlberg N Kristensen VN Borresen-Dale AL Saviranta P Perala M Kallioniemi O Protein lysate microarray analysis to identify microRNAs regulating estrogen receptor signaling in breast cancer cell lines Oncogene 2009 28 3926 3936 19684618 10.1038/onc.2009.241

[B63] Yoshimoto N Toyama T Takahashi S Sugiura H Endo Y Iwasa M Fujii Y Yamashita H Distinct expressions of microRNAs that directly target estrogen receptor alpha in human breast cancer Breast Cancer Res Treat 2011 130 331 339 21755340 10.1007/s10549-011-1672-2

[B64] Zhang H Su SB Zhou QM Lu YY [Differential expression profiles of microRNAs between breast cancer cells and mammary epithelial cells] Ai Zheng 2009 28 493 499 Article in Chinese 19624877

[B65] Manavalan TT Teng Y Appana SN Datta S Kalbfleisch TS Li Y Klinge CM Differential expression of microRNA expression in tamoxifen-sensitive MCF-7 versus tamoxifen-resistant LY2 human breast cancer cells Cancer Lett 2011 313 26 43 21955614 10.1016/j.canlet.2011.08.018 PMC3214732

[B66] Sobin LH Gospodarowicz MK Wittekind C Breast tumours TNM Online 2003 Hoboken, NJ: John Wiley & Sons

