# Supplementary material for: Serum microRNA expression as an early marker for breast cancer risk in prospectively collected samples from the Sister Study cohort
Source: Breast Cancer Res. 2013 May 24;15(3):R42. doi: 10.1186/bcr3428 (PMC3706791; doi:10.1186/bcr3428)
Supplement: Additional file 1 — Spearman correlation coefficient values for technical replicates of arrays. Three replicate serum samples from three women (nine samples in total) were processed and hybridized to arrays as described for samples in the main study. Spearman correlation coefficients were calculated for the three pairings of replicate samples for each woman and averaged. One array from Individual 1 appeared to be an outlier but was included in the results shown above. Exclusion of this array resulted in correlation coefficients of greater than 0.97 in all three categories of probes for Individual 1. [file bcr3428-S1.PDF]

	All Probes	All Human Probes	All Human Probes above threshold
Individual 1	0.884	0.873	0.815
Individual 2	0.953	0.947	0.942
Individual 3	0.949	0.941	0.936

Additional file 1. Spearman correlation coefficient values for technical replicates of arrays. Three replicate serum samples from 3 women (9 samples total) were processed and hybridized to arrays as described for samples in the main study. Spearman correlation coefficients were calculated for the three pairings of replicate samples for each woman and averaged. One array from Individual 1 appeared to be an outlier, but was included in the results shown above. Exclusion of this array resulted in correlation coefficients > 0.97 in all three categories of probes for Individual 1.
