# Supplementary material for: Serum microRNA expression as an early marker for breast cancer risk in prospectively collected samples from the Sister Study cohort
Source: Breast Cancer Res. 2013 May 24;15(3):R42. doi: 10.1186/bcr3428 (PMC3706791; doi:10.1186/bcr3428)
Supplement: Additional file 2 — Efficiency of the four polymerase chain reaction (PCR) assays. The efficiency of PCR amplifications for the normalization control and three target microRNAs (miRNAs) was calculated by using DART-PCR version 1.0. The average efficiency of each of three independent PCRs was similar across all four miRNAs. [file bcr3428-S2.PDF]

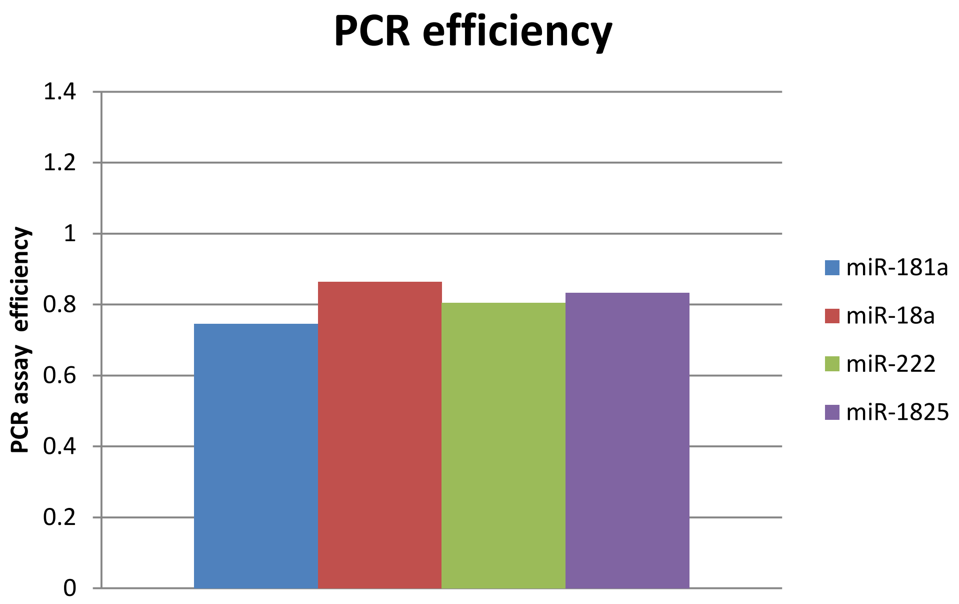

Additional file 2. Efficiency of the 4 PCR assays. The efficiency of PCR amplifications for the normalization control and 3 target miRNAs was calculated using DART-PCR version 1.0 [1]. The average efficiency of each of 3 independent PCR reactions was similar across all 4 miRNAs.

1.Peirson, S.N., J.N. Butler, and R.G. Foster, *Experimental validation of novel and conventional approaches to quantitative real-time PCR data analysis.* Nucleic Acids Res, 2003. **31**(14): p. e73.
