# Supplementary material for: Serum microRNA expression as an early marker for breast cancer risk in prospectively collected samples from the Sister Study cohort
Source: Breast Cancer Res. 2013 May 24;15(3):R42. doi: 10.1186/bcr3428 (PMC3706791; doi:10.1186/bcr3428)
Supplement: Additional file 3 — Quantitative reverse transcription-polymerase chain reaction (qRT-PCR) validation in 10 cases and controls. Serum levels of five cases and five controls were examined by using qRT-PCR for miR181a, miR18a, and miR-222. Box plots show normalized relative expression. Normalization was carried out by using the mean of miR-1825 and spiked in cel-39. The horizontal line represents the mean for each sample. [file bcr3428-S3.PDF]

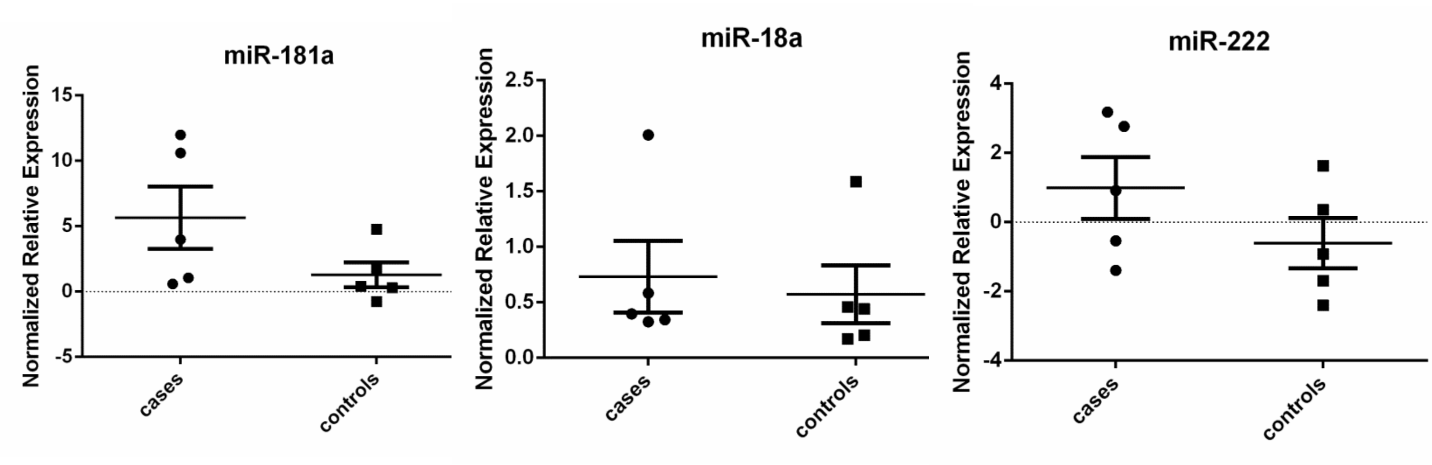

Additional file 3. qRT-PCR validation in 10 cases and controls. Serum levels of 5 cases and 5 controls were examined using qRT-PCR for miR-181a, miR-18a, and miR-222. Box plots show normalized relative expression. Normalization was done using the mean of miR-1825 and spiked in cel-39. Horizontal line represents the mean for each sample.
