# Supplementary material for: Serum microRNA expression as an early marker for breast cancer risk in prospectively collected samples from the Sister Study cohort
Source: Breast Cancer Res. 2013 May 24;15(3):R42. doi: 10.1186/bcr3428 (PMC3706791; doi:10.1186/bcr3428)
Supplement: Additional file 4 — Experimentally observed targets enriched for cancer and signaling pathways. Identified ingenuity pathway analysis (IPA) canonical pathways enriched by the experimentally observed targets of the 21 miRNAs differentially expressed between the cases and non-cases. The negative log (10) false discovery rate-corrected P values are shown. Note that this test has not corrected for possible dependencies across the mRNAs considered and that statistical significance may be overstated. HER-2, human epidermal growth factor receptor 2; ILK, integrin-linked kinase; PTEN, phosphatase and tensin homolog. [file bcr3428-S4.PDF]

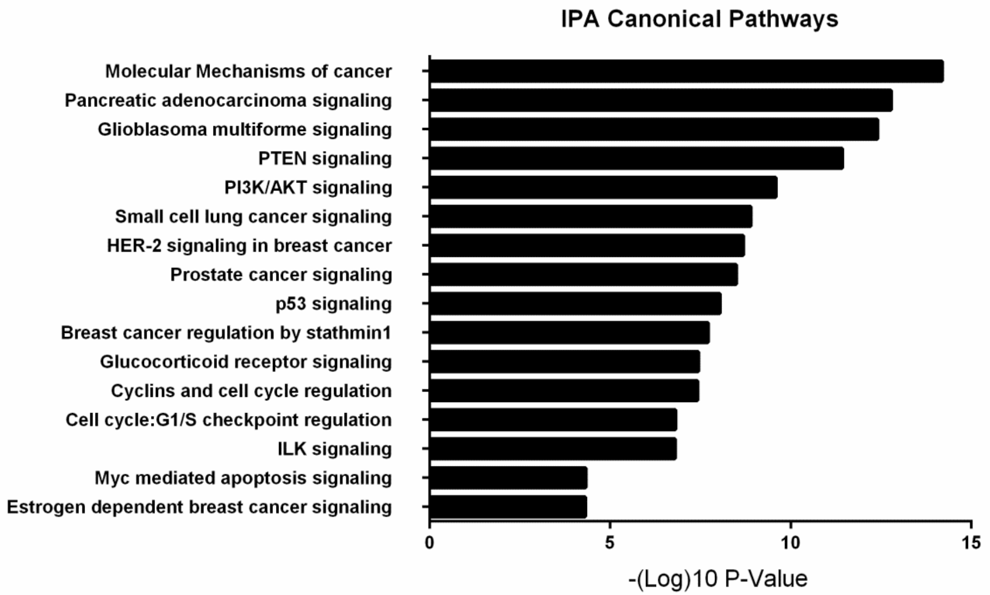

Additional file 4. Experimentally observed targets enriched for cancer and signaling pathways. Identified IPA Canonical Pathways enriched by the experimentally observed targets of the 21 miRNAs differentially expressed between the cases and non-cases. The negative Log (10) FDR corrected P-values are shown. Note that this test has not corrected for possible dependencies across the mRNAs considered, and statistical significance may be overstated.
